# Prospecting the antimicrobial and antibiofilm potential of *Chaetomium globosum* an endophytic fungus from *Moringa oleifera*

**DOI:** 10.1186/s13568-020-01143-y

**Published:** 2020-11-11

**Authors:** Navdeep Kaur, Daljit Singh Arora

**Affiliations:** Microbial Technology Laboratory, Department of Microbiology, Guru Nanak Dev University, Amritsar, 143005 India

**Keywords:** *Moringa oleifera*, Endophytes, Fungi, Antimicrobial, Antibiofilm, Biosafety

## Abstract

The current study prospects the antimicrobial potential of an endophytic fungus *Chaetomium globosum* which showed a wide spectrum antimicrobial activity against the tested pathogenic microorganisms. This is apparently the first report where *Chaetomium globosum* as an endophyte from *Moringa oleifera* showed antimicrobial potential and is optimized for physiochemical parameters to enhance the antimicrobial metabolites production. In the classical optimization yeast peptone dextrose medium, inoculum size of two discs, incubation period of 6 days, production temperature of 25 ºC and pH 7 was best supportive for optimal growth and antimicrobial activity whereas maltose and ammonium nitrate were the best carbon and nitrogen sources, respectively. The statistical optimization resulted in up to 1.33 fold increase in antimicrobial activity. Chloroform was found to be the best extractant. The chloroformic extract showed minimum inhibitory concentration ranging from 0.05 to 5 mg/ml and its microbicidal nature was established by viable cell count studies. The efficacy of the extract was also established in terms of post antibiotic effect which ranged from 2 to 20 h. The chloroformic extract exhibited the good antibiofilm potential and was also found to be biosafe. The clinical relevance of the study was justified as it showed good antimicrobial efficacy against some resistant clinical isolates, too.

## Introduction

The emergence and spread of antimicrobial resistance by pathogenic microorganisms to commercial drugs is a noteworthy issue endured by mankind and has turned into an important concern throughout the globe (Rajeswari et al. 2016). This problem has risen due to the certain factors, such as over and misuse of antibiotics, poor hygienic conditions, increased number of patients with weakened immune system and delay in diagnosis of diseases (Bockstael and Aerschot 2008). Therefore, it is important to search out new and potent antimicrobial agents to overcome the problems raised by various resistant pathogenic organisms by scouting untapped resources (Xing et al. 2011).

The human health has been much dependent on plants since ages, because of the wide spread belief that ‘green medicines’ are healthier and safer than the synthetic ones (Newman and Cragg 2007). Another reason for the consideration of natural resources in the field of healthcare is the increasing incidences of multidrug resistant organisms arising from the consistent use of synthetic medicines for the treatment of various ailments (Selvamohan et al. 2012). In the last few years, endophytic microorganisms have occupied a centre stage in the field of antimicrobials. Microbial species present in inter- and intracellular spaces of plant tissues without causing apparent damage are known as endophytes (Pavithra et al. 2012). These may help their host to synthesize the bioactive metabolites such as alkaloids, quinones, terpenoids, isocoumarins, steroids, lignans, phenols, and lactones (Strobel et al. 2004). So endophytes became a great source of research for the development of novel drugs for medical and agricultural purposes (Li et al. 2012; Sharma et al. 2016).

Plants used for the medicinal purposes since ages have been considered for the search of endophytes with the novel and bioactive compounds, as the bioactive metabolites of the plants may be derived from the endophytes residing in it (Kusari et al. 2013). *Moringa oleifera* is one such magic plant which has been used in the treatment of various pathogenic diseases in the ancient medicinal system (Singh et al. 2009). Due to the medicinal properties of *Moringa oleifera*, it was explored to find out the bioactive endophytes and *Chaetomium globosum* is one such endophytic fungus that is able to produce metabolites with bioactive potential. Many endophytic strains of *Chaetomium globosum* have been reported to show their antimicrobial potential (Samaga et al. 2014). It is known to produce various bioactive secondary metabolites belonging to diverse structural types of chaetoglobosins, epipolythiodioxopiperazines, azaphilones, xanthones, anthraquinones, chromones, depsidones, terpenoids and steroids (Tran et al. 2010; Li et al. 2011). Various physiochemical parameters have been optimized for one such endophytic fungus *C. globosum* to enhance the antimicrobial potential. Other in vitro studies such as minimum inhibitory concentration (MIC), viable cell count studies (VCC) and post antibiotic effect (PAE) have also been carried out, along with the antibiofilm potential of the chloroformic extract of *C. globosum*. To provide clinical credence to the study, the biosafety of the chloroformic extract was evaluated by Ames test and MTT assay and has also been tested for its effectiveness against the clinically resistant strains of MRSA and one strain of *Enterococcus* sp.

## Material and methods

### Identification of fungus

The endophytic fungus DSE 72 used throughout the study was isolated from *Moringa oleifera* seed (Arora and Kaur 2019) and its molecular identification was done by National Fungal Culture Collection of India (NFCCI), Agharkar Research Institute, Pune, India. The ITS sequence obtained was deposited in NCBI GenBank with accession number (MN416318). The isolate *Chaetomium globosum* (DSE 72) has been deposited in National Fungal Culture Collection of India (NFCCI), Agarkar Research Institute, Pune, India vide accession number NFCCI 4822.

### Test organisms

The reference strains of bacteria: *Staphylococcus aureus* (MTCC-740) *Staphylococcus epidermidis* (MTCC-435), *Klebsiella pneumoniae* 1 (MTCC-109), *Klebsiella pneumoniae* 2 (MTCC-530), *Escherichia coli* (MTCC-119), *Shigella flexneri* (MTCC-1457), *Pseudomonas aeruginosa* (MTCC-741), *Salmonella typhimurium* 1 (MTCC-98), *Salmonella typhimurium* 2 (MTCC-1251) and *Enterococcus faecalis* (MTCC-439) and two yeast strains: *Candida albicans* (MTCC 227) and *Candida tropicalis* (MTCC 230), used for testing their sensitivity to endophytic fungal extract, were obtained from Microbial Type Culture Collection (MTCC), Institute of Microbial Technology (IMTECH), Chandigarh, India. A clinical isolate, i.e., Methicillin-resistant *Staphylococcus aureus* (MRSA) was procured from Post Graduate Institute of Medical Education and Research (PGIMER), Chandigarh, India.

Other clinical isolates which included, eleven strains of MRSA (DSACI 01 to DSACI 11) and one strain of *Enterococcus* species (DSACI 12) were procured from Department of Microbiology, Government Medical College and Hospital, Amritsar.

### Preparation of extract and its screening for antimicrobial activity

Four mycelial discs (8 mm diameter) of *C. globosum* grown on Yeast Glucose Agar (YGA) plates were used to inoculate 50 ml of YG broth containing (g/l): yeast extract 3, peptone 5, dextrose 10, and pH 5.5 and incubated at 25 °C as stationary cultures for 5 days. The harvested broth was assayed for screening the antimicrobial activity by agar well diffusion assay (ADA) where the diameter of resultant zone of inhibition, if any, was measured (Arora and Kaur 2019; Bauer et al. 1966).

### Optimization of antimicrobial activity of *C. globosum*

Different physiochemical parameters were optimized for the fungus to enhance its antimicrobial potential where the culture broth samples obtained for each parameter were tested for their antimicrobial activity against seven microorganisms (*Enterococcus faecalis*, *Staphylococcus aureus, Staphylococcus epidermidis, Klebsiella pneumoniae* 1, *Salmonella typhimurium* 2, *Candida albicans* and MRSA) which were earlier found to be sensitive to culture broth of *C. globosum* by agar well diffusion assay. All the experiments were done in triplicates.

Antimicrobial activities of the culture broth samples obtained after 5 days of incubation at 25 °C under static conditions with different discs (2–10 discs) against the selected microorganisms were determined by ADA. To select the suitable basal medium, the different media used were Czapek dox’s broth (CD), Potato dextrose broth (PD), Yeast glucose broth (YG), Sabouraud dextrose broth (SD) and Yeast peptone dextrose broth (YPDS). Similarly to check the effect of incubation period the fungal strain was grown for 30 days at 25 °C at static conditions. Initially activity was taken at the interval of 3 days upto 18 days and then at an interval of 6 days upto 30 days of incubation. Optimum temperature was determined by growing the fungus at different temperatures (15, 20, 25, 30, 35, 40, 45 °C). To determine the effect of pH the fungus was grown at different pH values (3–9) at 25 °C under static conditions for 5 days.

To study the effect of different carbon sources, dextrose in the production medium (YPDS) of *C. globosum* was replaced by sucrose, (carboxy methyl cellulose (CMC), maltose, starch, xylose and fructose. Similarly, the influence of nitrogen sources on metabolites production was monitored by replacing the yeast extract with other nitrogen sources like malt extract, sodium nitrate, ammonium nitrate, ammonium chloride, potassium nitrate, ammonium sulphate, urea and casein (Arora and Kaur 2019).

### Statistical optimization using Placket Burman design (PBD) and Response surface methodology (RSM)

P-B design was used for the screening of significant medium components which affect the production of antimicrobial metabolites. In this, the major components of the basal medium i.e. ammonium sulphate, peptone, maltose and starch, were screened and the ones showing the significant effect were further optimized by RSM using central composite design (CCD) as discussed earlier (Arora and Kaur 2019; Jose et al. 2013).

### Determination of best organic extractant

The extraction of culture broth of *C. globosum* was done with different organic solvents viz. chloroform, ethyl acetate, hexane, and butanol. Two phase metabolite extraction was done and the organic layer was evaporated using rotatary evaporator. The dried material so obtained was weighed and suspended in diluent i.e. 30% DMSO and subjected to antimicrobial screening against the twelve reference organisms and thirteen clinical isolates by agar well diffusion assay.

### Minimum inhibitory concentration (MIC)

MIC values for chloroformic extract of *C. globosum* were determined by agar dilution method using different concentrations (0.001% to 0.5%) from the stock solution (27.2 mg/ml). The concentration showing inhibition in terms of visible microbial growth was considered as MIC (Wiegand et al. 2008).

### Viable cell count studies (VCC) and post antibiotic effect (PAE)

The antimicrobial action of chloroformic extract i.e. microbistatic/cidal was ascertained in terms of time taken by the compound to induce complete killing of pathogenic microorganism. The stock solution at its 2× MIC was mixed with equal volume of activated bacterial culture which was further serially diluted to 10^–3^ using suitable broth medium (Arora et al. 2016).

PAE is important to determine the efficacy of any antimicrobial agent. It is the persistent suppression of bacterial growth after their brief exposure (1 or 2 h) to an antimicrobial agent. The PAE of the chloroformic extract was studied as discussed earlier (Arora and Onsare 2014).The stock solution of chloroformic extract at its 2× MIC was mixed with each test organism. The calculation of PAE is based on the equation PAE = T – C where T is the time for the count in the test culture to increase 1 log 10 cfu/ml above the count observed immediately after drug removal and C represent the time for the count of the untreated control to increase by 1log 10 cfu/ml.

## Antibiofilm potential of *Chaetomium globosum*

### Screening for biofilm formation by test organisms

Chloroformic extract obtained from *C. globosum* was tested for its antibiofilm potential against three organisms (*S. aureus, K. pneumoniae* 1 and *C. albicans*). Initial screening was carried out by tube method (Christensen et al. 1982) with slight modifications. A loopful of activated cultures were inoculated into 10 ml each of nutrient broth for bacterial and yeast malt broth for yeast and incubated for 24 h at 37 °C and 25 °C, respectively. The culture media from the tubes were then discarded, thereafter the tubes were washed with phosphate buffer saline (PBS pH 7.4) and dried before staining with 0.1% crystal violet. Further, the tubes were washed with deionised water to remove the extra stain. Air dried tubes were then observed for biofilm formation. The visible lining of the biofilm around the bottom and the walls showed the positive results. To establish the antibiofilm potential of chloroformic extract of *C. globosum*, the following assays were carried out.

### Inhibition of initial cell attachment assay

For each test organism, three sets of dispense i.e. test, positive control and experimental control were raised in microtitre plate. For test, 100 µl of chloroformic extract (at its 1× MIC) was added to the wells. In a same manner for positive control standard antibiotics (1 mg/ml) were added along with experimental control (broth medium) in triplicates. The same volume (100 µl) of microbial suspension of *S. aureus, K. pneumoniae* 1 and *C. albicans* were added into the wells of test and positive control and final volume per well was 200 µl. After incubation for 24 h at 37 °C for bacteria and at 25 °C for yeast, the inhibition potential of the extract was calculated as biofilm biomass obtained in comparison with experimental control (untreated cells) as described in crystal violet assay and expressed as percentage inhibition as given below.

### Disruptive potential of chloroformic extract on preformed biofilms

The disruptive effect of chloroformic extract of *C. globosum* was carried out as described earlier (Onsare and Arora 2015) with slight modifications. For the formation of biofilm, 100 µl of the inoculum was dispensed into each well of the microtitre plate and incubated for 24 h at 37 °C for bacteria and at 25 °C for yeast for cell attachment. After that, supernatant was discarded and 100 µl of the chloroformic extract or standard antibiotic were added to the wells having the preformed biofilm to make a final volume of 200 µl. whereas for the negative controls, the fresh broth was added instead of extract or antibiotic and incubated for 24 h.

After the incubation period the biomass was estimated by crystal violet assay where the wells were decanted and washed with sterile water to get rid of unattached cells. The plate was first air-dried and then dried at 60 °C in the oven for 45 min. Further, the staining was done with 100 µl of 0.1% crystal violet and incubated for 15 min. Then again the plate was washed with water to eliminate the non adsorbed cells. Further the wells were destained by using 125 µl of absolute ethanol and it was transferred to a new plate for the estimation of biofilm density and the absorbance was taken at 590 nm and compared with that of negative control.

Percentage inhibition = 100−[{OD of test or positive control well / OD of negative control well} × 100].

### Estimation of metabolic viable cells using XTT assay

The metabolic activity of biofilms produced by pathogenic microorganisms was demonstrated by XTT assay (Arora and Mahajan 2019). In this method, tetrazolium salt (XTT) was reduced to formazan derivative by the viable cells which was then quantified spectrometrically. The XTT solution (1 mg/ml) was prepared by dissolving it into the PBS buffer and was then filter sterilized and stored at − 70 °C. 10 mM of menadione was prepared by mixing it with acetone and filter sterilized. In the next step, 2.5 ml of thawed XTT solution was mixed with 2.5 µl and 20 µl of menadione for bacteria and yeast, respectively. The preformed biofilms were treated with the chloroformic extract and standard antibiotics as discussed earlier. After washing the wells 200 µl of menadione –XTT working solution was added into the well and incubated in dark for 2 h. The 100 µl from each well was dispensed into the fresh wells and the colour change was observed by microtitre plate reader at 490 nm. The percentage of viable cells was determined in comparing the mean absorbance values of test wells with that of the negative controls.

## Biosafety evaluation of *C. globosum* by Ames test and MTT assay

### Ames test

Ames test was performed by plate incorporation method, the test strain was grown in nutrient broth at 37 °C for 24 h and then diluted upto 10^–3^ dilution. To perform the mutagenecity test 36.6 μl of activated culture of *Salmonella typhimurium* (MTCC 1251) was mixed with 36.6 μl of chloroformic extract equivalent to 2× MIC and added to 5 ml of top agar having 0.5 mM histidine–biotin mixture (1:1 ratio) volume equivalent to the test strain. The mixture was poured onto glucose minimal agar plates. Sodium azide and sterilized distilled water were used as a positive and negative control respectively (Mortelmans and Zeiger 2000).

### MTT assay

The toxicity levels of chloroformic extract was worked out using sheep red blood cells (procured from local butcher shop, Amritsar) by MTT [3-(4, 5-dimethylthiazol-2-yl)-2, 5-diphenyl tetrazolium bromide] assay (Arora and Onsare 2014) where the extract was used at its 2× MIC for the test. The absorbance was taken at 590 nm on the microplate reader. The wells with untreated cells served as control.

## Results

### Identification of fungus

The molecular identification of the selected isolate (DSE 72) was done by National Fungal Culture Collection of India (NFCCI), Agarkar Research Institute, Pune, India. Genomic DNA was isolated in pure form from the cultures. The ITS region of rDNA was successfully amplified using fungal universal primers ITS4 and ITS5. After this, the sequencing of the fungal isolate was carried out and it has been identified as *Chaetomium globosum* and the sequence has been deposited in Genbank under the accession number (MN416318). The isolate *Chaetomium globosum* (DSE 72) has been deposited in National Fungal Culture Collection of India (NFCCI), Agarkar Research Institute, Pune, India vide accession number NFCCI 4822.

### Screening of *C. globosum* for antimicrobial activity by agar well diffusion assay

The extracellular culture broth of *Chaetomium globosum* was effective against eight out of thirteen tested organisms with the inhibition zone ranging from 16.3 to 21.5 mm. Among the gram positive bacteria, it was effective against the *S. aureus* and MRSA while among the gram negative bacteria *K. pneumoniae* 1 was the most sensitive. The yeast strain *C. albicans* was the least sensitive organism and *C. tropicalis* was completely resistant (Additional file 1: Table S1).

### Optimization of the antimicrobial activity of *C. globosum*

**Fig. 1:**
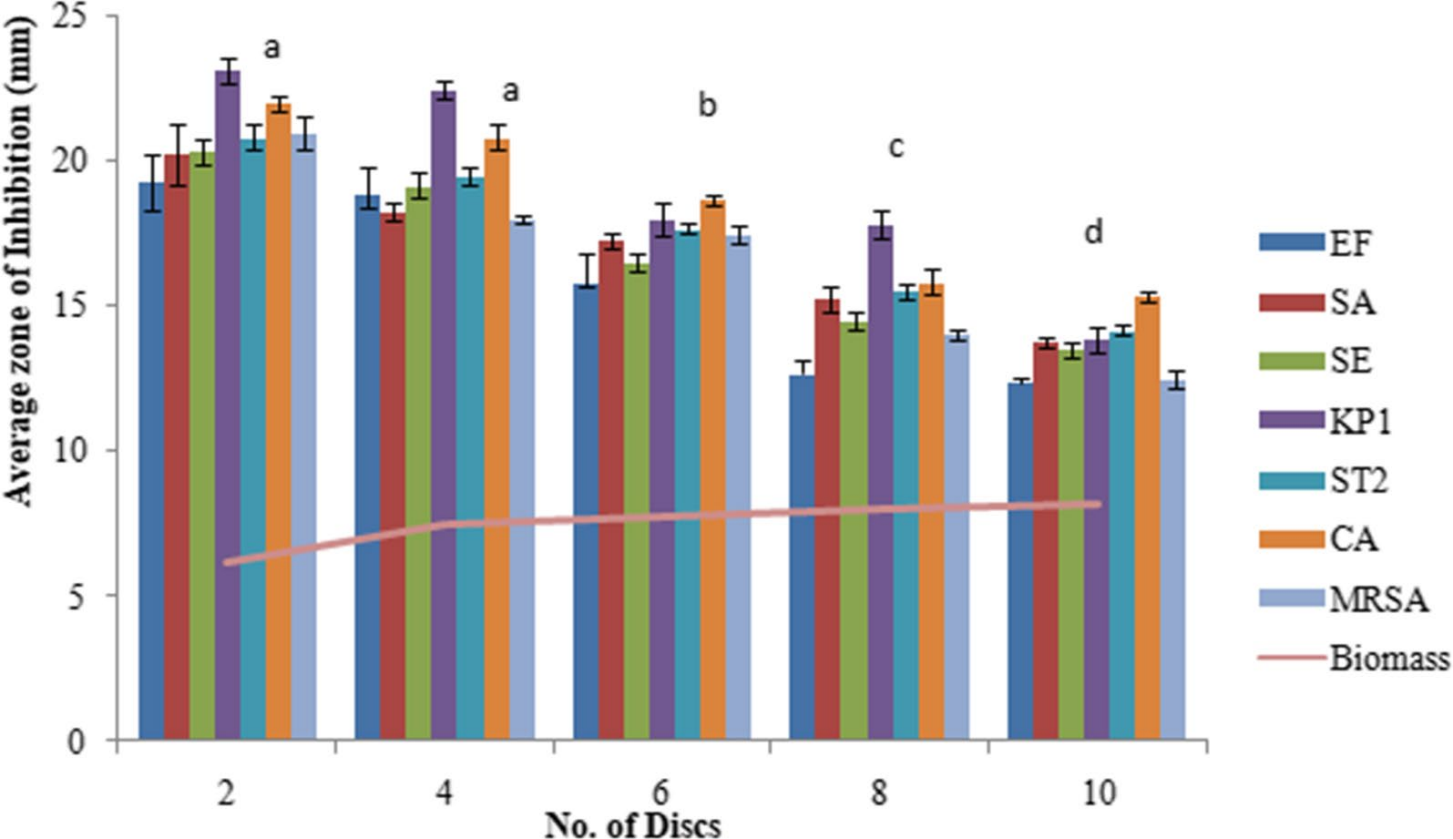
Effect of inoculum size on antimicrobial activity of *C. globosum*. EF, *Enterococcus faecalis; *SA, *Staphylococcus aureus*; SE, *Staphylococcus epidermidis*; KP1, *Klebsiella pneumoniae* 1; ST2, *Salmonella typhimurium* 2; CA, *Candida albicans* MRSA, Methicillin-resistant *Staphylococcus aureus*. *Values are given as mean ± SE. Different letters (a to d) between the columns are significantly different (post hoc tukey Test, p ≤ 0.05)

**Fig. 2:**
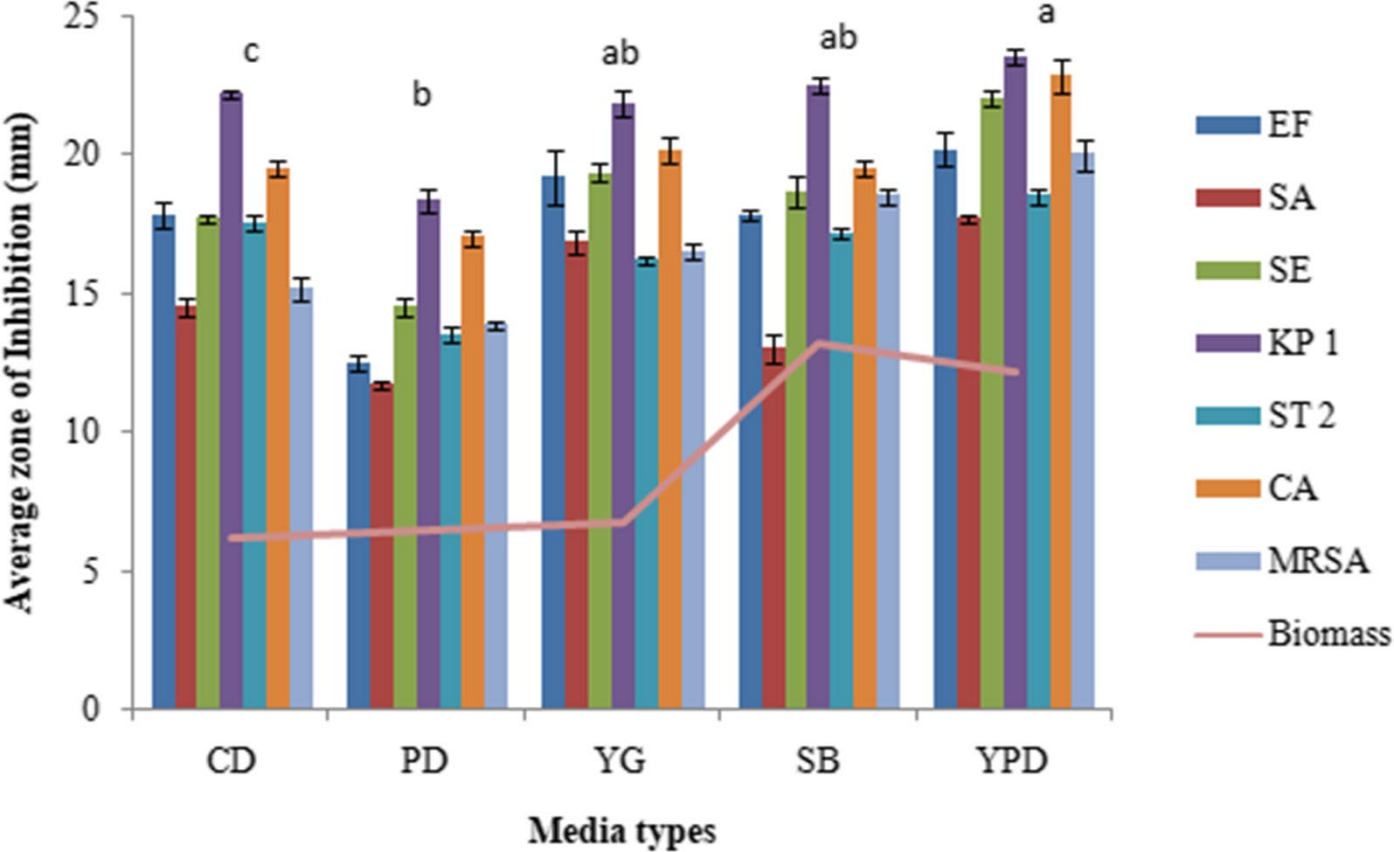
Effect of different growth media on antimicrobial activity of *C. globosum.* EF, *Enterococcus faecalis;*SA, *Staphylococcus aureus*; SE, *Staphylococcus epidermidis*; KP1, *Klebsiella pneumoniae* 1; ST2, *Salmonella typhimurium* 2; CA, *Candida albicans* MRSA, Methicillin-resistant *Staphylococcus aureus.* *Values are given as mean ± SE. Different letters (a to c) between the columns are significantly different (post hoc tukey Test, p ≤ 0.05)

**Fig. 3:**
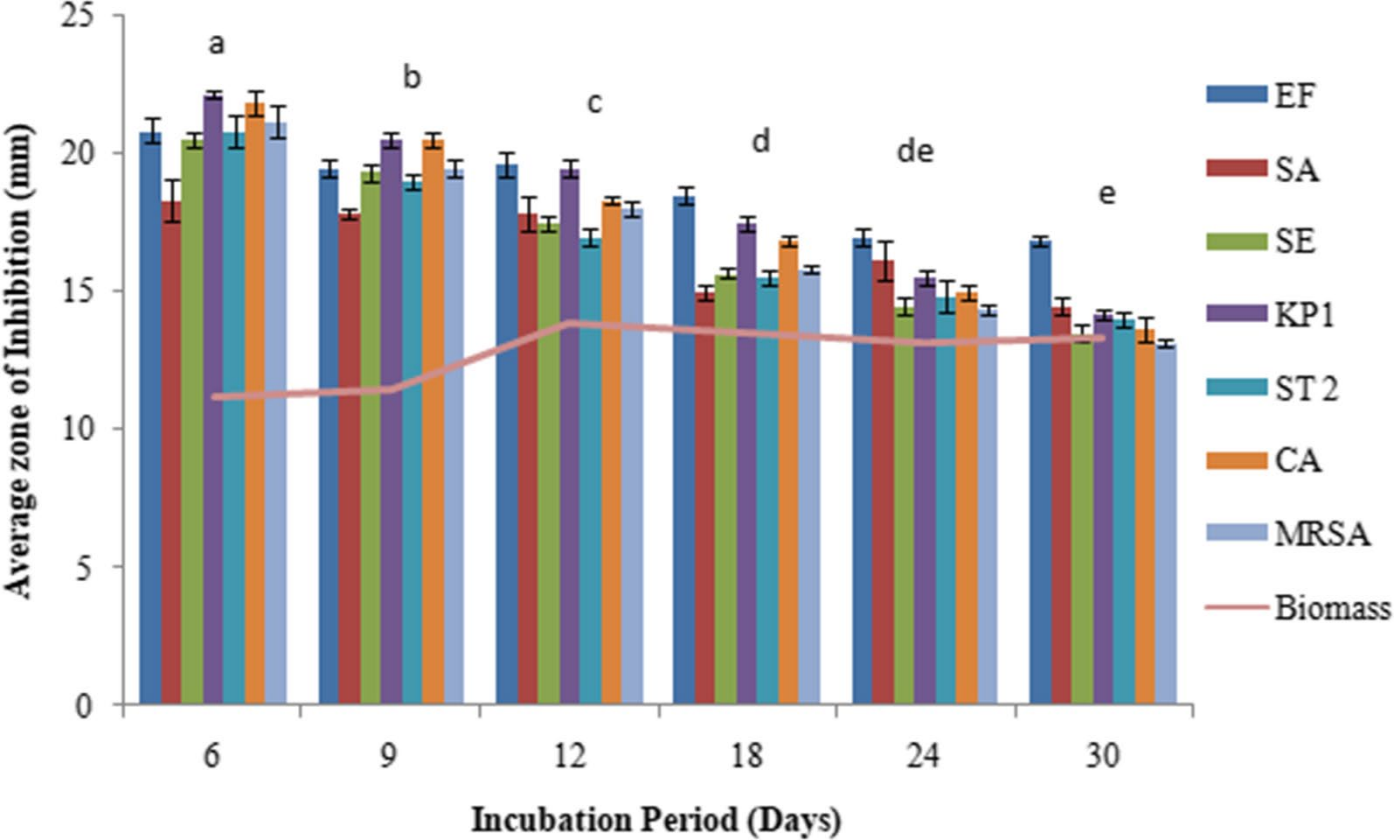
Effect of different incubation period on antimicrobial activity of *C. globosum*. EF, *Enterococcus faecalis; *SA, *Staphylococcus aureus*; SE, *Staphylococcus epidermidis*; KP1, *Klebsiella pneumoniae* 1; ST2, *Salmonella typhimurium* 2; CA, *Candida albicans* MRSA, Methicillin-resistant *Staphylococcus aureus.* *Values are given as mean ± SE. Different letters (a to e) between the columns are significantly different (post hoc tukey Test, p ≤ 0.05)

**Fig. 4:**
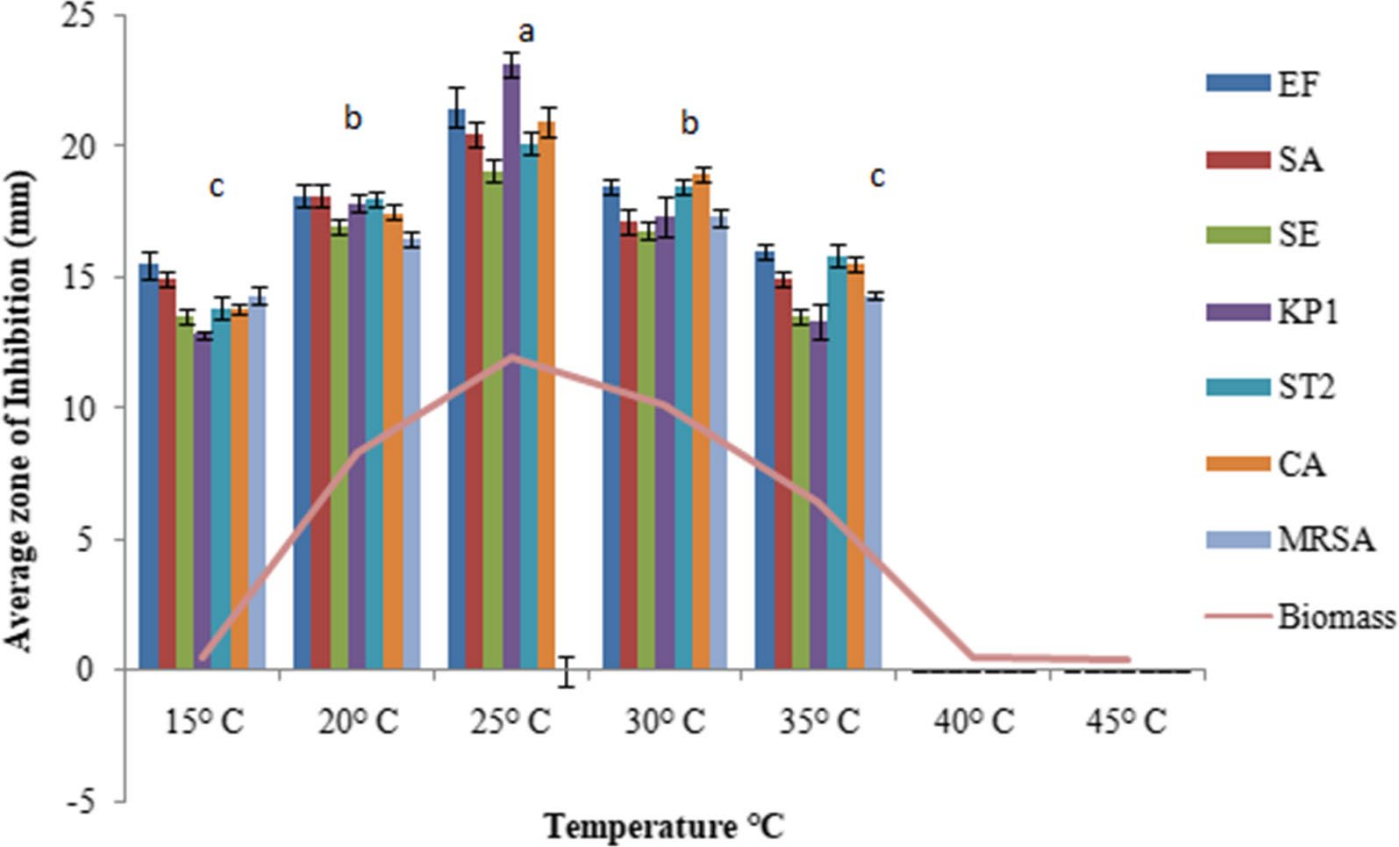
Effect of temperature on antimicrobial activity of *C. globosum.* EF, *Enterococcus faecalis;*SA, *Staphylococcus aureus*; SE, *Staphylococcus epidermidis*; KP1, *Klebsiella pneumoniae* 1; ST2, *Salmonella typhimurium* 2; CA, *Candida albicans* MRSA, Methicillin-resistant *Staphylococcus aureus*. *Values are given as mean ± SE. Different letters (a to c) between the columns are significantly different (post hoc tukey Test, p ≤ 0.05)

**Fig. 5:**
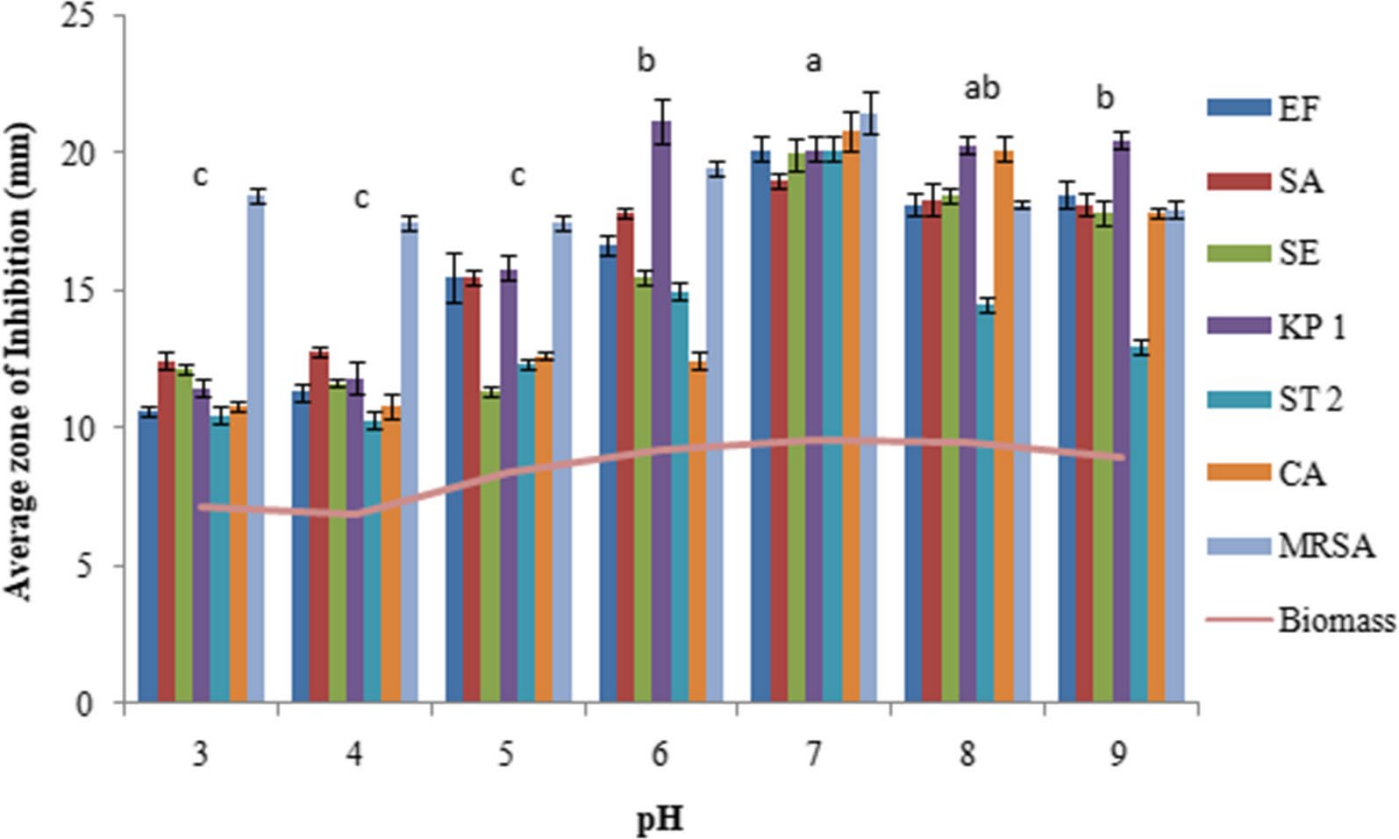
Effect of pH on antimicrobial activity of *C. globosum*. EF, *Enterococcus faecalis; *SA, *Staphylococcus aureus*; SE, *Staphylococcus epidermidis*; KP1, *Klebsiella pneumoniae* 1; ST2, *Salmonella typhimurium* 2; CA, *Candida albicans* MRSA, Methicillin-resistant *Staphylococcus aureus*. *Values are given as mean ± SE. Different letters (a to c) between the columns are significantly different (post hoc tukey Test, p ≤ 0.05)

The extracellular culture broth of *C. globosum* inoculated with 2 discs showed the maximum antimicrobial activity with an inhibition zone (IZ) ranging from 19.3 to 23.1 mm for different microorganims followed by the inoculum size of 4 discs (IZ ranging from 18 to 22.5 mm). Thereafter not much change in activity was observed when using 6 discs and the activity rather declined on using 8 to 10 discs. (Fig. 1). YPDS broth medium was the best to sustain maximum antimicrobial activity followed by YG and SD broth media. However, maximum biomass was produced in SD broth medium (Fig. 2). Six days of incubation period was optimum for antimicrobial activity which almost remained constant up to 12th day of incubation, thereafter it declined. Maximum biomass was produced on 12th day (Fig. 3). The optimum temperature for antimicrobial activity was 25 °C. At temperature extremes of 15 °C and 35 °C very low activity was observed and no fungal growth was observed above 35 °C (Fig. 4). The optimum pH was 7 with an inhibition zone (IZ) ranging from 18.5 to 20 mm which showed slight variations up to pH 9. Very low activity was observed at acidic pH. Maximum biomass was produced at pH 6 which remained stable up to pH 9 (Fig. 5).

**Fig. 6:**
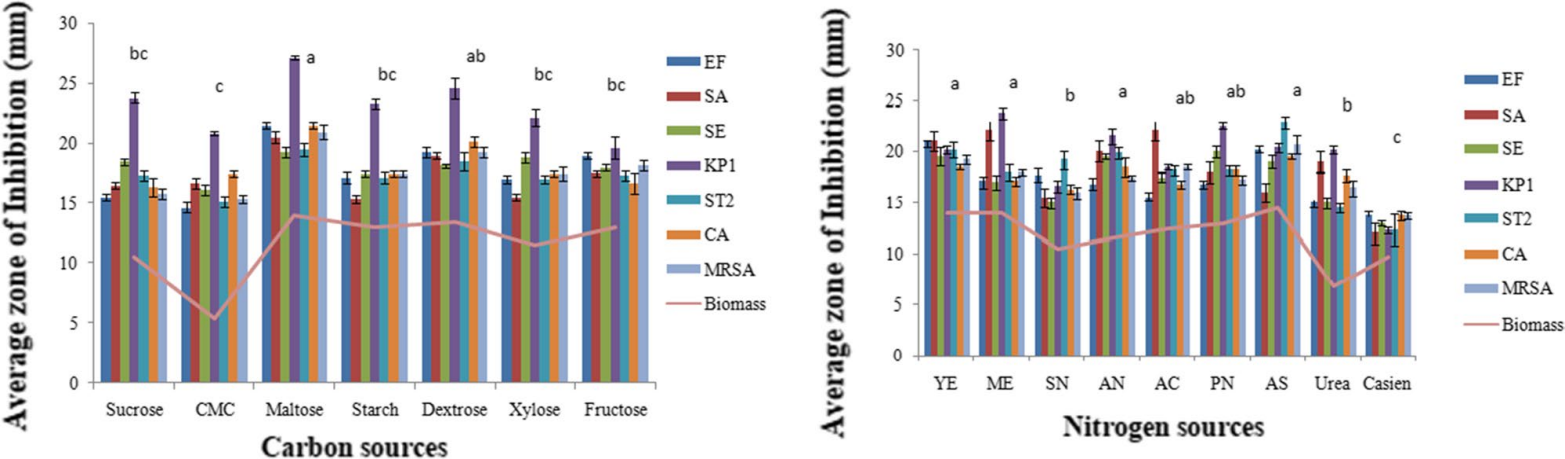
Effect of different carbon sources and nitrogen sources on antimicrobial activity of *C. globosum*. EF, *Enterococcus faecalis; *SA, *Staphylococcus aureus*; SE, *Staphylococcus epidermidis*; KP1, *Klebsiella pneumoniae* 1; ST2, *Salmonella typhimurium* 2; CA, *Candida albicans* MRSA, Methicillin-resistant *Staphylococcus aureus.* *Values are given as mean ± SE. Different letters (a to c) between the columns are significantly different (post hoc tukey Test, p ≤ 0.05)

Maltose was the best carbon source to support the maximum antimicrobial activity closely followed by dextrose. However, the latter supported the maximum biomass. On the other hand, ammonium sulphate was the best nitrogen source for maximum antimicrobial activity closely followed by yeast extract, malt extract, potassium nitrate and ammonium nitrate. The maximum biomass was however obtained in ammonium sulphate, yeast extract and malt extract basal medium (Fig. 6). A significant difference (p < 0.05) between the various parameters was observed by the one way ANOVA followed by post hoc tukey test.

### Statistical optimization using Placket Burman design (PBD) and Response surface methodology (RSM)

**Fig. 7:**
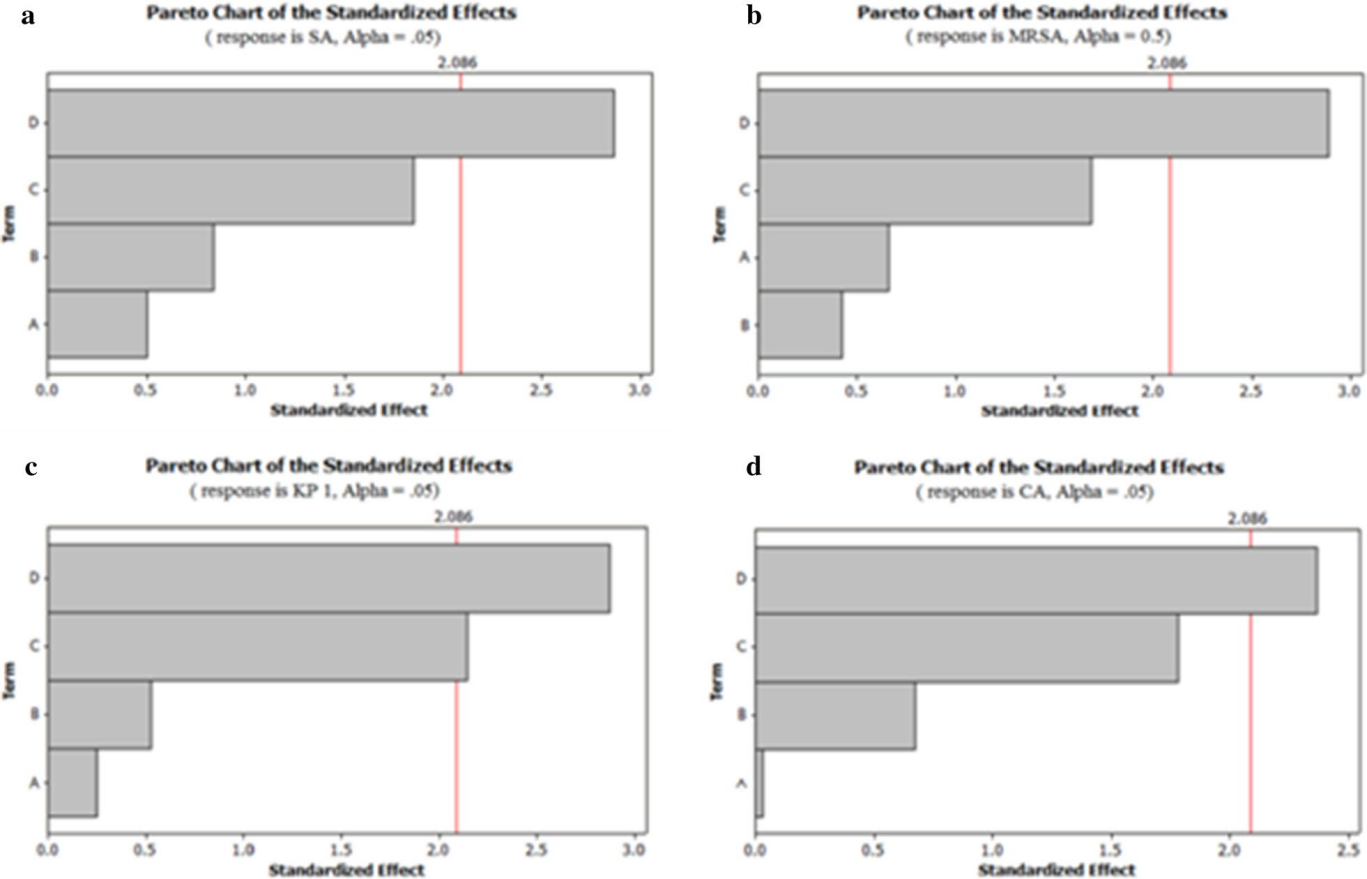
Pareto charts of **a** *S. aureus*, **b** MRSA, **c** *K. pneumoniae* 1, **d** *C. albicans.* *D-Starch, C-Maltose, B-Peptone, A-Ammonium sulphate

As tested by PBD, starch, maltose and peptone showed a significant influence on antimicrobial activity of *C. globosum*. These variables showed 90% confidence levels which indicate their significant contribution than those of other media components. The effect of the components was also confirmed by the Pareto graphs where the higher effects were shown in the upper position which then progressed down to the lower effects (Fig. 7).

In the next step, the concentration of the selected variables was optimized by RSM using central composite design. The significance of model was examined by the P-values and the analysis of variance (ANOVA) is presented in Table 1 which showed the (P < 0.05) means model is statistically significant, at confidence level of 95%. The coefficient of determination R2, were found to be 87.2%, 90%, 93% and 85.3% for *S. aureus*, MRSA, *K. pneumoniae* 1, *C. albicans* respectively.

**Table 1:** Analysis of variance (ANOVA) for the quadratic model

Source	DF	Seq. SS				Adj. MS				F value				P value			
		SA	MRSA	KP 1	CA	SA	MRSA	KP 1	CA	SA	MRSA	KP 1	CA	SA	MRSA	KP 1	CA
Regression	9	50.854	51.255	81.627	30.492	4.650	5.695	9.069	3.388	7.59	10.00	14.69	6.46	0.002	0.001	0.01	0.004
Residual	10	7.445	5.695	6.173	5.246	7.445	5.695	0.617	0.524								

*DF* degree of freedom, *SS* sum of squares, *MS* mean square; R2 = 95%

**Fig. 8:**
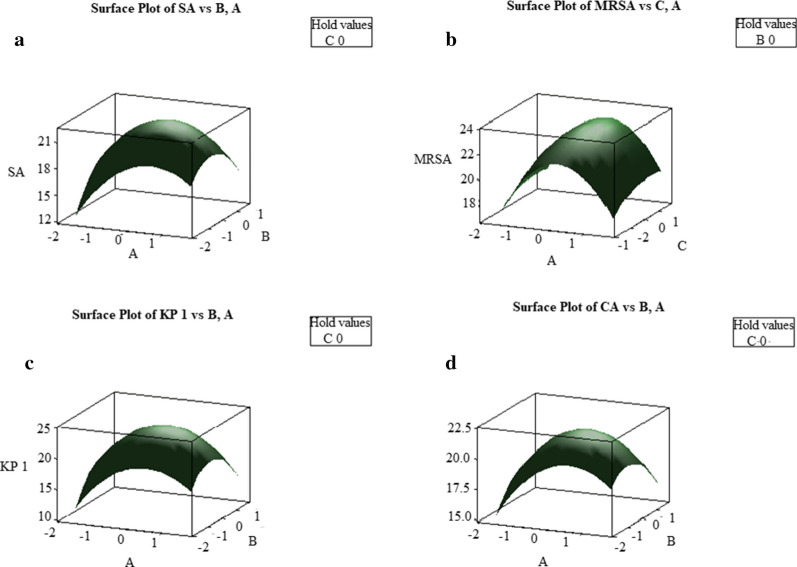
Response surface plots of **a** *S. aureus*, **b** MRSA, **c** *K. pneumoniae 1*, **d** *C. albicans*

The squared effect of peptone was significant in case of *C. albicans*. The interaction between the starch and peptone was positive in case of all the organisms and the other interactions were negative. The predicted model has also been presented in the form of three dimensional (3D) response surface graphs (Fig. 8). The optimal values of variables obtained from 3D plots were, starch = 15–12.5, maltose = 5–7.5 and peptone = 5–7.5 (g/l). Further by using optimized medium, validation of the statistical results was done which proved that experimental values agreed with the predicted values and increased the antimicrobial activity by 1.29-folds for *S. aureus*, 1.33-folds for MRSA and *K. pneumoniae* 1- and 1.2-folds against *C. albicans*.

### Determination of best extractant for antimicrobial activity

Chloroform was found to be the best extractant for antimicrobial activity followed by ethyl acetate, hexane and butanol. The organisms such as *K. pneumoniae* 2, *S. flexneri* and *S. typhimurium* 1 which were resistant to extracellular culture broth were found to be sensitive to organic extract. However *E.coli, Pseudomonas aeruginosa* and *C. tropicalis* remained resistant. The data was also compared with standard antibiotics (Additional file 1: Table S2).

### Minimum inhibitory concentration (MIC)

The MIC of the chloroformic extract of *C. globosum* was strain specific and it ranged from 0.05 to 5 mg/ml. *K. pneumoniae* 1 and *S. epidermidis* were the most sensitive organisms showing a MIC of 0.05 mg/ml followed by *S. aureus* and *S. typhimurium* 2 (0.1 mg/ml). *E. faecalis* and *C. albicans* showed the MIC of 0.5 mg/ml. However, MRSA showed an MIC of 1 mg/ml whereas *K. pneumoniae* 2, *S. flexneri* and *S. typhimurium* 1 showed the highest MIC of 5 mg/ml.

### Viable cell count studies (VCC) and Post antibiotic effect (PAE)

In VCC studies, *E. faecalis*, K. *pneumoniae* 1 and *S. typhimurium* 1 got completely killed in 4 h whereas *S. typhimurium* 2, MRSA and *K. pneumoniae* 2 took 8 h for complete killing. S*. flexneri* and *C albicans* got killed in 10 h. However *S. epidermidis* took 12 h for complete killing. Chloroformic extract was found to be bactericidal in nature as no regrowth occurred even after 24 h of incubation in any organism. Post antibiotic effect is persistent suppression of bacterial growth after their brief exposure (1 or 2 h) to an antimicrobial agent. PAE against different microorganisms ranged from 2 to 20 h where *K. pneumoniae* 1 showed the longest PAE of 20 h followed by MRSA and *S. aureus* showed the PAE of 8 h.

### The effect of chloroformic extract of *C. globosum* on the initial cell attachment of the organisms

**Fig. 9:**
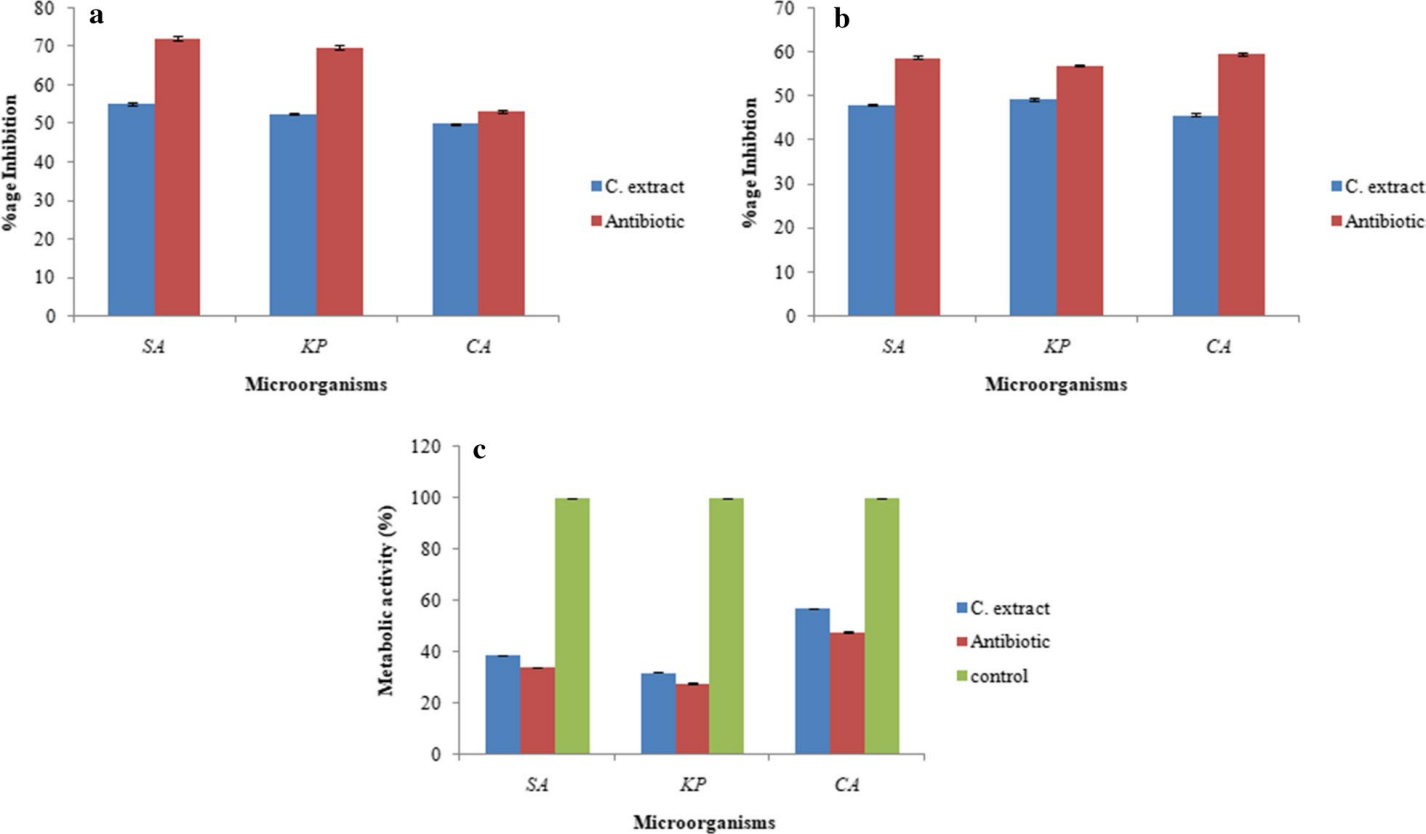
Effect of chloroformic extract on **a** Initial cell attachment, **b** Performed biofilms, **c** Metabolic activities of treated biofilms. *SA, *Staphylococcus aureus*; KP*, Klebsiella pneumoniae* 1 and CA, *Candida albicans*

The chloroformic extract of *C. globosum* demonstrated its strong antibiofilm activity i.e. 55.2% inhibition of *S. aureus*, 52.6% of *K. pneumoniae* 1 and 50% of *C. albicans* in their initial cellular attachment. In case of *C. albicans*, the inhibition was comparable with the amphotericin B (53.3%) (Fig. 9a).

### Effect of chloroformic extract of *C. globosum* on the disruption of preformed biofilm

As the developed biofilms are more resistant to antimicrobial agents, the chloroformic extract resulted in 48% inhibition of *S. aureus*, 49.3% inhibition of K*. pneumoniae* 1 and 45.8% inhibition of *C. albicans* (Fig. 9b).

### Metabolic activity of preformed biofilm by chloroformic extract of *C. globosum* by XTT assay

In the above experiments, crystal violet assay quantifies the biofilm biomass but does not reveal viability of sessile cells. But in this experiment XTT (tetrazolium salt) reduced to an orange color formazan crystals to indicate the quantity of viable cells which was measured colorimetrically at 490 nm. Here the chloroformic extract slows down the respiratory potential of test organisms where *K. pneumoniae* 1 showed only 32% metabolic activity followed by *S. aureus* which showed 38.9% metabolic activity and *C. albicans* showed 56.8% metabolic activity of biofilms. On the other hand antibiotics gentamicin and amphotericin B showed the 34%, 27% and 47.7% metabolic activity in case of *S. aureus, K. pneumoniae* 1 and *C. albicans*, respectively. Thus the chloroformic extract showed a good antibiofilm potential (Fig. 9c).

## Biosafety evaluation of *C. *globosum by Ames test and MTT assay

The non mutagenic nature of the chloroformic extract was demonstrated by Ames test where no revertant colonies were obtained whereas the positive control i.e. sodium azide showed numerous colonies. In addition to this, the cytotoxicity profile of chloroformic extract evaluated by MTT assay showed 94.8% viable cells as compared to that of untreated control, thus clearly establishing the non mutagenic as well as non cytotoxic profile of the chloroformic extract.

### Antimicrobial activity of chloroformic extract of *C. globosum* against some clinical isolates

To validate and highlight the importance of the study, the chloroformic extract of *C. globosum* was tested for antimicrobial activity against some clinical isolates. All the clinical isolates were found to be sensitive with IZ ranging from 21.16 to 31.83 mm where DSACI 09 was the most sensitive organism and DSCI 03 was found to be least sensitive (Additional file 1: Table S3).

## Discussion

The burning problem of increasing microbial resistance has led the researchers to look for the novel sources of antimicrobial agents to control the drug resistant pathogens. Keeping this in mind, an endophytic fungal isolate *C. globosum*, obtained from *Moringa oleifera* has been studied for its antimicrobial potential. In the preliminary screening by ADA, the extracellular culture broth of *C. globosum* showed the antimicrobial efficacy against 53.84% of the tested microbial strains. It was highly effective against a common public health associated infectious agent i.e. *K. pneumoniae* 1, which causes severe infections of the urinary tract, bloodstream, and intra abdominal infections (Yu et al. 2019). The effectiveness of *C. globosum* against the clinical isolates of MRSA, further endorses the relevance of the study against resistant pathogens. *C. globosum* has been reported earlier as an endophyte for various biological activities in a few studies (Dissanayake et al. 2016; Samaga et al. 2014; Selim et al.^2014^), but apparently this is the first report of *C. globosum* as an endophyte from *Moringa oleifera* exhibiting the antimicrobial activity. In the classical optimization, the inoculum size of 2 discs was found to be optimum which is in consonance with earlier studies where the authors used different size of discs (2 to 12 mm) and reported 6 mm discs to be the best (Jain and Pundir 2011). Medium optimization is important for growth and synthesis of primary and secondary metabolites and the best way to obtain highest possible product of interest. YPD was found to be the best supporting medium which goes well with earlier observations (Kaur and Arora 2015). The optimum period of incubation was 5 days. In an earlier study, on endophytic fungus *Chaetomium* sp., an incubation period of 14 days has been reported for its antimicrobial activity (Fatima et al. 2016). The antimicrobial activity was optimal at pH 7 and is in line with the previous studies on production of antibacterial compound by endophytic fungi *Nigrospora* sp. (Sandey et al. 2015). Maximum antimicrobial activity at 25 °C is in consonance with the previous studies reporting it to be the optimum temperature for secondary metabolite production and growth by *Aspergillus* strain TSF146 (Bhattacharyya and Jha 2011) The presence of carbon and nitrogen sources is considered to be important to affect the production of bioactive metabolites (Chen et al. 2010). In the present study, maltose supported the best antimicrobial activity. Similarly maximum production of antimicrobial metabolite was reported in the presence of sucrose as carbon source by endophytic fungus *Arthrinium* (Ramos and Said 2011). Ammonium nitrate was the best nitrogen source which is in consonance with earlier studies on endophytic fungus *Hypocrea* spp. where it helps in the synthesis of secondary metabolite (Gogoi et al. 2008). The information about nutritional conditions regulating the metabolism helps to maximize the production of the antimicrobial metabolites which can be further exploited for various pharmaceutical purposes. The statistical optimization resulted in 1.33-fold increase in antimicrobial activity, in line with the previous reports on studies carried out with *Penicillium* sp. (Kaur et al. 2015).

The extraction of secondary metabolites with different organic solvents showed chloroform to be the best. The suitability of the organic solvent highly depends upon the nature of the bioactive metabolites. The antimicrobial activity was significantly better against *S. aureus, E. faecalis* and *C. albicans* as compared to silver nanoparticles synthesized using endophytic fungus, *Cryptosporiopsis ericae* PS4 (Devi and Joshi 2014). Low MIC of fungal chloroformic extract (0.05 mg/ml to 0.5 mg/ml) marks its importance and corroborates the earlier reports where MIC of endophytic fungi ranged between 0.156 to 0.625 mg/ml (Mahadevamurthy et al. 2016). The time kill studies revealed the bactericidal nature of the chloroformic extract, which will be useful for pharmaceutical purposes (Thammawat et al. 2017). PAE studies are finding importance to work out the dosing schedule of antimicrobial administration in a more scientific way (Devi and Joshi 2014) and in the present studies, the chloroformic extract retained its effectiveness for variable times, *K*. *pneumoniae* 1 showed the longest PAE of 20 h which clearly endorses the MIC and VCC studies indicating it to be the most sensitive organism. The antibiofilm potential demonstrated by chloroformic extract further highlight the importance of the study as the efficiency of the extract against *C. albicans* on its initial cell attachment compared well with the amphotericin B. Also the percentage inhibition in preformed biofilms revealed that it is difficult to kill the adhered cells (Veloz et al. 2019).The non cytotoxic profiling of the chloroformic extract revealed it to be biosafe as demonstrated by MTT assay and Ames test. In order to provide further credence to the study and scientific validation in the public health system, the chloroformic extract of *C. globosum* was randomly tested for its antimicrobial activity against the selected clinical isolates. The results demonstrated reasonably good antimicrobial activity against eleven drug resistant strains of MRSA and one strain of *Enterococcus* sp. Thus, the study can substantially contribute to the battle against the prevailing antibiotic resistance.

The study concludes that chloroformic extract of *C. globosum* exhibited the significant broad spectrum antimicrobial as well as antibiofilm activity. Moreover, the fungal extract was also found to biosafe, but before taking the issue further for drug development, the in vivo experimentation needs to be done to ensure the biosafety of the extract as well as the compounds. To provide more confidence to the user and justify the study, antimicrobial testing can be extended further against more reference strains as well as clinical isolates of pathogenic microorganims.

## Supplementary information

**Additional file 1: Table S1.** Antimicrobial activity of *C. globosum* against some potential pathogens. **Table S2.** Antimicrobial activity of chloroformic extract of *C. globosum*. **Table S3.** Antimicrobial activity of chloroformic extract of *C. globosum* against some clinical isolates of MRSA (DSACI01 to DSACI011) and one strain of *Enterococuss* sp. (DSACI012).
