# Supplementary material for: Prospecting the antimicrobial and antibiofilm potential of Chaetomium globosum an endophytic fungus from Moringa oleifera
Source: AMB Express. 2020 Nov 11;10:206. doi: 10.1186/s13568-020-01143-y (PMC7658282; doi:10.1186/s13568-020-01143-y)
Supplement: Supplementary file 1 — Additional file 1: Table S1. Antimicrobial activity of C. globosum against some potential pathogens. Table S2. Antimicrobial activity of chloroformic extract of C. globosum. Table S3. Antimicrobial activity of chloroformic extract of C. globosum against some clinical isolates of MRSA (DSACI01 to DSACI011) and one strain of Enterococuss sp. (DSACI012). [file 13568_2020_1143_MOESM1_ESM.docx]

**Table S1: Antimicrobial activity of *C. globosum* against some potential pathogens:**

| **Organisms** | **Zone of Inhibition (mm)** |
| --- | --- |
| *Enterococcus faecalis* | 18.8±0.92 |
| *Staphylococcus aureus* | 21.1±0.60 |
| *Staphylococcus epidermidis* | 17.2±.033 |
| *Escherichia coli* | **-** |
| *Klebsiella pneumoniae* 1 | 21.5±0.60 |
| *Klebsiella pneumoniae* 2 | **-** |
| *Pseudomonas aeruginosa* | **-** |
| *Shigella flexneri* | **-** |
| *Salmonella typhimurium* 1 | - |
| *Salmonella typhimurium 2* | 19±0.28 |
| *Candida albicans* | 16.3±0.33 |
| *Candida tropicalis* | **-** |
| MRSA | 17.5±0.28 |

The values were expressed as mean±SE (n=3)

**Table S2: Antimicrobial activity of chloroformic extract of *C. globosum***

|  | **Inhibition zone (mm)** | | |
| --- | --- | --- | --- |
| **Organisms** | ***C. globosum*** | **Gentamicin** | **Chloramphenicol** |
| *E.faecalis* | 21.33±0.33 | 27.5±0.28 | 26.5±0.28 |
| *S. aureus* | 20.33±0.33 | 34.5±0.28 | 26.66±0.33 |
| *S. epidermidis* | 24±0.57 | 26.1±0.44 | 27.33±0.44 |
| *K.pneumoniae* 1 | 28.16±0.44 | 33.33±0.60 | 38.5±0.28 |
| *K.pneumoniae* 2 | 22.1±0.60 | 34.33±0.44 | 27.66±0.16 |
| *S.flexneri* | 21.16±0.72 | 27.83±0.44 | 30.16±0.44 |
| *S.typhimurium* 1 | 23.13±0.69 | 32.33±0.44 | 32.83±0.44 |
| *S.typhimurium* 2 | 20.66±0.33 | 30.5±0.5 | 26.5±0.28 |
| *C. albicans* | 24.5±0.76 | 35.5±0.5^a^ | ND |
| MRSA | 22.83±0.16 | 39.83±0.44 | 33.5±0.28 |

ND not done

a Amphotericin B

**Table S3:** **Antimicrobial activity of chloroformic extract of *C. globosum* against some clinical isolates of MRSA (DSACI01 to DSACI011) and one strain of *Enterococuss sp*. (DSACI012)**

| **Organisms** | **Chloroformic extract** |
| --- | --- |
| DSACI01 | 26±0.28 |
| DSACI02 | 22.16±0.44 |
| DSACI03 | 21.66±0.16 |
| DSACI04 | 24.5±0.28 |
| DSACI05 | 27.5±0.28 |
| DSACI06 | 27.33±0.60 |
| DSACI07 | 27.83±0.16 |
| DSACI08 | 27.5±0.28 |
| DSACI09 | 31.83±0.44 |
| DSACI010 | 23.83±0.44 |
| DSACI011 | 22.5±0.28 |
| DSACI012 | 26.5±0.28 |

The values were expressed as mean±SE (n=3)
